# Effect of Thrombopoietin Receptor Agonist Romiplostim on the Ionizing Radiation‐Induced Premature Aging

**DOI:** 10.1002/prp2.70203

**Published:** 2025-12-01

**Authors:** Masaru Yamaguchi, Tokuhisa Hirouchi, Yoshiaki Sato, Ikuo Kashiwakura

**Affiliations:** ^1^ Department of Radiation Science Hirosaki University Graduate School of Health Sciences Hirosaki Japan; ^2^ Department of Radiobiology Institute for Environmental Sciences Rokkasho Japan; ^3^ Norris Comprehensive Cancer Center University of Southern California California Los Angeles USA

**Keywords:** mouse model, radiation‐induced premature aging, romiplostim, total body radiation

## Abstract

Environmental stressors, such as ionizing radiation, accelerate aging by causing DNA damage and triggering pathways that lead to cell cycle arrest, apoptosis, and subsequent inflammation. The thrombopoietin receptor agonist romiplostim (RP), which is used as a clinical treatment for chronic idiopathic thrombocytopenic purpura and aplastic anemia, is known to be promising in reducing radiation‐induced tissue damage. In this study, we established a mouse model of radiation‐induced premature aging to evaluate the potential of RP in ameliorating this process. Female C57BL/6JJcl mice were subjected to total body irradiation with various irradiation schedules. Mice irradiated with 5 Gy every 4 weeks (total dose of 10 Gy over 2 months) showed a significant aging phenotype, including graying hair and elevated serum aging markers (CDKN2A/p16^INK4a^, tumor necrosis factor‐α (TNF‐α), and C‐reactive protein), compared with sham‐irradiated controls. RP was intraperitoneally administered to the mouse model (10 μg/kg weekly or 50 μg/kg every 4 weeks). Treatment significantly reduced TNF‐α levels by 15% and the area of graying body hair by 70%. Although bone marrow cell recovery was incomplete, spleen cell counts were significantly restored (2‐fold) by 50 μg/kg RP, and SA‐β‐gal activity, a marker of cellular senescence, was also significantly suppressed by approximately 15%. These findings suggest that RP may partially ameliorate radiation‐induced premature aging, providing a basis for future research addressing health issues associated with aging and radiation exposure.

## Graphical abstract

Mice irradiated with 5 Gy every 4 weeks (total dose of 10 Gy over 2 months) showed a significant aging phenotype, but thrombopoietin receptor agonist Romiplostim (RP) treatment partially ameliorate radiation‐induced premature aging.

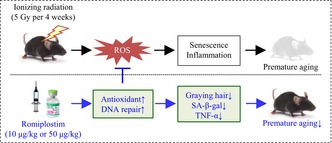

**Abbreviations**
BMCs: bone marrow cells
CRP: C‐reactive protein
IFN‐γ: interferon‐γ
IL‐6: interleukin‐6
ROS: reactive oxygen species
RP: romiplostim
SA‐β‐Gal: senescence‐associated β‐galactosidase
SPCs: spleen cells
TBI: total body irradiation
TNF‐α: tumor necrosis factor‐α

## 1 Introduction

Aging is a multifactorial process involving irreversible functional and structural changes, influenced by both genetic and environmental stressors [1]. Ionizing radiation causes severe molecular damage, including DNA double‐strand breaks, leading to cell cycle arrest, apoptosis, and cellular senescence, as well as increased expression of p16^INK4a^ and p21^CIP1/WAF1^ [2]. It also triggers oxidative stress, mitochondrial dysfunction, and chronic inflammation via the overproduction of reactive oxygen species (ROS), thereby disrupting tissue homeostasis [3]. In human cells and mouse models, ionizing radiation increases the senescence‐associated β‐galactosidase (SA‐β‐Gal)‐positive cells, persistent accumulation of phosphorylated histone H2AX at serine 139 (γ‐H2AX), and enhanced secretion of pro‐inflammatory cytokines such as interleukin‐6 (IL‐6) and tumor necrosis factor‐α (TNF‐α), which are hallmarks of radiation‐induced premature aging [2, 3]. These effects pose significant health risks in real‐world settings, including long‐term spaceflight, occupational exposure in medical and nuclear fields, and cancer radiotherapy, all linked to elevated risks of aging‐related diseases due to oxidative stress and inflammation [4]. Understanding the molecular mechanisms of radiation‐induced premature aging and developing countermeasures to prevent or ameliorate this process will be vital for medicine, industrial safety, and geriatric care.

Romiplostim (RP), a fusion protein analog of thrombopoietin, regulates platelet production and is approved for treating chronic idiopathic thrombocytopenic purpura and aplastic anemia. Recently, it has also gained attention as a medical countermeasure for hematopoietic acute radiation syndrome. In our previous studies, administration of RP to mice exposed to lethal doses of radiation not only promoted hematopoiesis in multiple organs, such as the spleen and lung, but also reduced DNA damage and apoptotic cell death, thereby improving the survival rate [5]. Notably, a slight survival benefit was observed at 10 μg/kg RP or higher, with the most pronounced mitigative effect achieved at 50 μg/kg, demonstrating a clear dose‐dependent response. The 50 μg/kg dose corresponds to approximately 50 times the standard clinical dose used for chronic idiopathic thrombocytopenic purpura (1 μg/kg) and 5 times that used for aplastic anemia (10 μg/kg), yet remains within the nontoxic range [5, 6, 7]. Furthermore, RP has been shown to induce the expression of antioxidant enzymes through activation of the Nrf2‐Keap1 (nuclear factor erythroid 2‐related factor 2 and Kelch‐like ECH‐associated protein 1) pathway [7], suggesting an indirect ameliorative effect on radiation‐induced premature aging via alleviation of oxidative stress and cell damage. In this study, we established a mouse model of radiation‐induced premature aging to investigate the ameliorative effects of RP. Given that RP is already clinically available, it represents an attractive strategy that can significantly reduce the time and cost required for drug development.

## 2 Materials and Methods

### 2.1 Ethics Statement

All procedures related to animal studies were approved by the Animal Research Committee of Hirosaki University (approval number: G17001) and were conducted in accordance with the ARRIVE guidelines. In this study, every effort was made to minimize the number and suffering of animals used in the study and to comply with current animal welfare regulations.

### 2.2 Experimental Design

**FIGURE 1:**
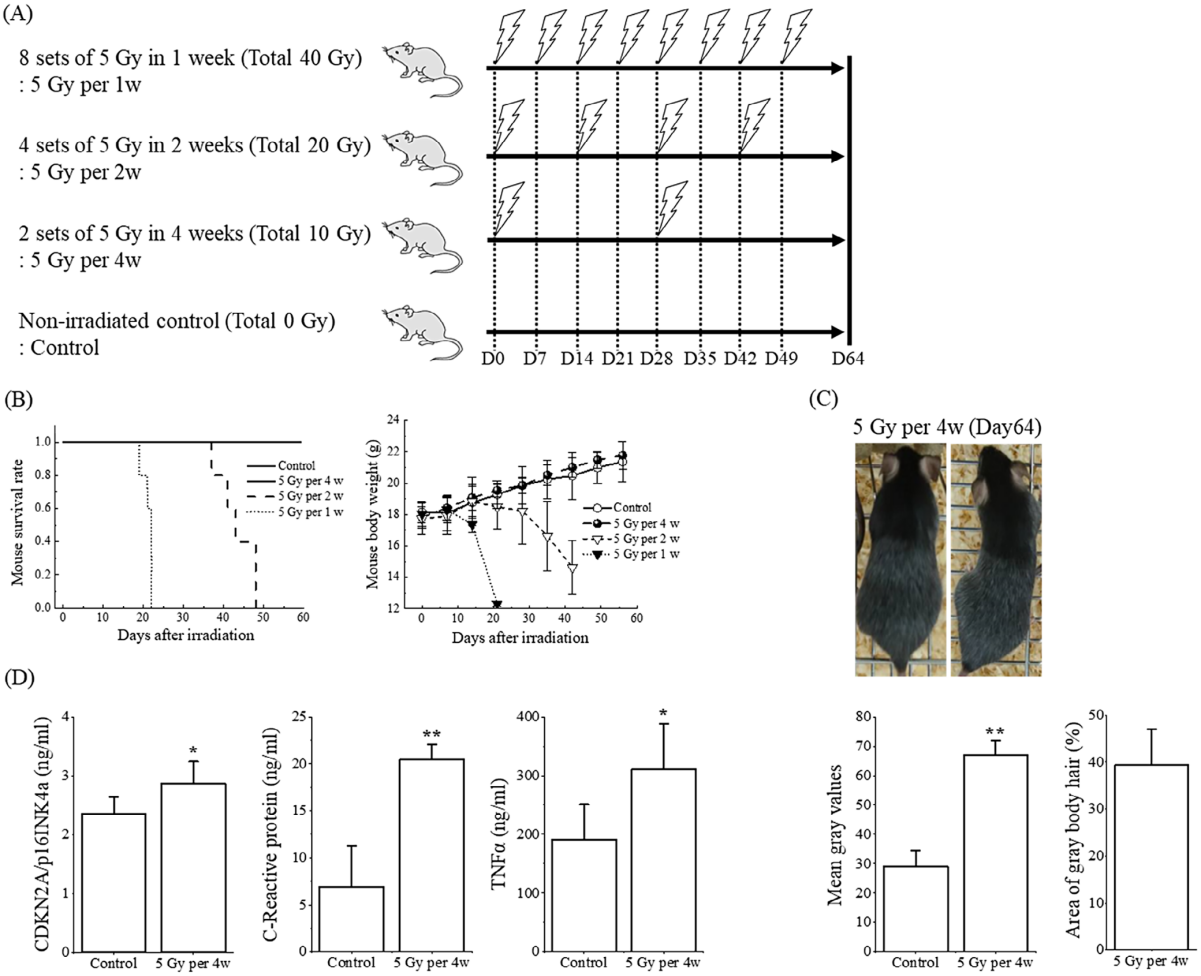
Establishment of a radiation‐induced premature aging mouse model. (A) Mice were subjected to sham and various total body γ irradiations and analyzed on day 64 (each *N* = 5). (B) Mouse survival rate and body weight. (C) Representative hair graying images, mean gray value, and percentage of graying hair. (D) Concentrations of serum CDKN2A/p16^INK4a^, CRP, and TNF‐α. Data are shown as the mean ± standard deviation. Statistical comparisons were performed between the two groups, with * *p* < 0.05 and ** *p* < 0.01.

**FIGURE 2:**
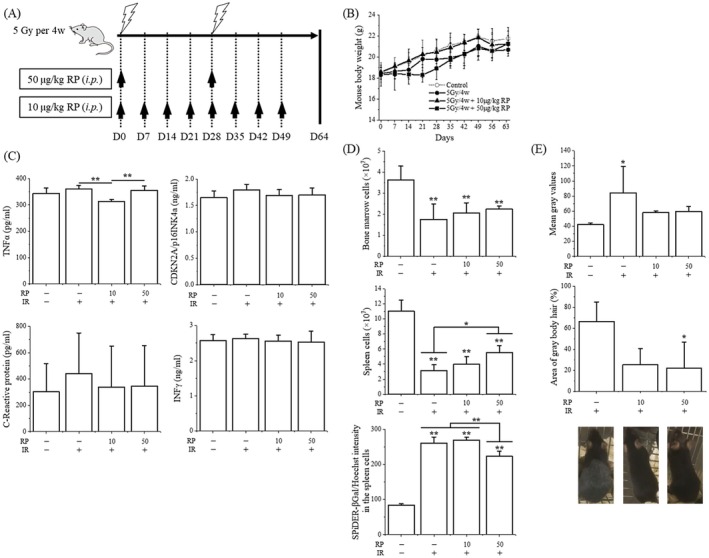
Ameliorative effect of RP on radiation‐induced premature aging. (A) Mice were irradiated with 5 Gy total body X‐ray twice, intraperitoneally administered RP at 10 μg/kg eight times or 50 μg/kg twice, and analyzed on day 64 (each *N* = 5). (B) Mouse body weight. (C) Concentrations of serum CDKN2A/p16^INK4a^, CRP, TNF‐α, and IFN‐γ. (D) Viable BMC and SPC counts, and expression of SA‐β‐gal in SPCs. (E) Representative hair graying images, mean gray value, and percentage of graying hair. Data are shown as the mean ± standard deviation. Statistical comparisons were performed between multiple groups, with * *p* < 0.05 and ** *p* < 0.01.

Seven‐week‐old inbred female C57BL/6JJcl mice were obtained from Clea Japan (Tokyo, Japan). Individual identification was performed by ear punch. Mice were randomly housed in sterile polycarbonate cages with stainless steel mesh lids and paper bedding, with a maximum of five mice per cage. They were allowed to acclimate for 1 week in clean cages before X‐ray irradiation. The animal facility was maintained at 23°C with 50% relative humidity and a 12‐h light/dark cycle. Immediately after arrival, mice were provided ad libitum access to CLEA Rodent Diet CE‐2 and tap water from Aomori Prefecture. Cages were changed weekly to ensure hygienic conditions. Using these acclimatized mice, we established a radiation‐induced premature aging model (Figure 1) and investigated the ameliorative effects of drug administration (Figure 2). Total body irradiation (TBI) was performed with either ^137^Cs γ‐ or X‐rays (150 kV, 20 mA, 0.5‐mm aluminum and 0.3‐mm copper filters) using a Gammacell 40 Exactor (Best Theratronics, Ottawa, Canada) or an MBR‐1520R X‐ray generator (Hitachi Medical, Tokyo) at a dose rate of 0.69 or 1.0 Gy/min, respectively. Mice were irradiated in four regimens: eight times of 5 Gy weekly (5 Gy per 1w; total 40 Gy), four times of 5 Gy biweekly (5 Gy per 2w; total 20 Gy), two times of 5 Gy every 4 weeks (5 Gy per 4w; total 10 Gy), or sham‐irradiation (Control; total 0 Gy) (Figure 1A). Mice were monitored once daily at a fixed time to assess general health, including body weight, food intake, activity levels, and coat condition. TBI mice were classified in the highest distress category, as they were expected to experience unavoidable severe stress and prolonged pain. Humane endpoints were considered when mice displayed signs of distress, abnormal appearance, or sudden weight loss exceeding 20%. However, no animals were excluded from the study due to reaching humane endpoints. All procedures, including mouse care, irradiation, sample collection, and analysis, were performed by different researchers to minimize bias. The number of mice used for each experiment is indicated in the figure legends.

### 2.3 Mitigator Treatment

The thrombopoietin receptor agonist RP (Romiplate, Kyowa Kirin, Tokyo) was administered intraperitoneally to TBI mice weekly for 8 times or once every 4 weeks for two times (Figure 2A). The dose of RP was 10 or 50 μg/kg of body weight, respectively [5, 6, 7].

### 2.4 Evaluation of Graying Hair

The graying hair due to TBI was analyzed using ImageJ version 1.54 g. For each photographed mouse, the region of interest was manually defined along the body contour, and only the region of interest was analyzed. Images were split into grayscale channels and thresholder to distinguish black and gray hairs based on the histogram peaks of pixel intensity. From the peaks representing black and gray hairs, the mean gray value and the area of graying relative to the total area of the region of interest were calculated.

### 2.5 Sample Collection

On day 64 after initial TBI, mice were fully anesthetized with isoflurane (Powerful Isoful, Zoetis, London, UK) and then euthanized by cervical dislocation. Peripheral blood was collected from each mouse, clotted for 1 h at room temperature, and centrifuged at 1200 × g for 10 min, and the supernatant was collected as serum components. Bone marrow cells (BMCs) were harvested from both femurs by flushing with ethylenediaminetetraacetic acid‐phosphate‐buffered saline, containing 0.5% bovine serum albumin. The spleens were mashed on a mesh filter, and spleen cells (SPCs) were harvested using Hank's balanced salt solution. Both cell suspensions were filtered through a 45‐μm strainer and hemolyzed in Gey's salt solution for red blood cell lysis. Viable cells were counted using a hemocytometer (BurkerTurk; Sunlead Glass, Saitama, Japan) with the trypan blue dye exclusion method (Sigma‐Aldrich; St. Louis, MO, USA).

### 2.6 Measurement of Inflammation and Aging Markers

The concentrations of serum C‐reactive protein (CRP), CDKN2A/p16^INK4a^, TNF‐α, and interferon‐γ (IFN‐γ) were analyzed using the mouse C‐reactive protein ELISA kit (Helica Biosystems, Scottsdale, AZ, USA), mouse CDKN2A ELISA kit (LifeSpan BioSciences, Seattle, WA, USA), and mouse TNF‐α / IFN‐γ ELISA kit (Proteintech, Rosemont, IL, USA), respectively. In addition, SA‐β‐gal expression in SPCs was analyzed using the Cellular Senescence Plate Assay Kit (SPiDER‐βGal, Dojindo, Kumamoto, Japan) according to the manufacturer's instructions.

### 2.7 Statistical Analyses

The statistical analyses were performed using the Excel 2019 software program (Microsoft, Redmond, WA, USA) with the Statcel 4 (OMS, Tokyo). Comparisons between two groups used the Student's *t* test, the Welch's *t* test, or the Mann–Whitney *U* test. Multiple groups were compared using one‐way ANOVA and Tukey–Kramer or Bonferroni/Dunn tests. A *p* value of < 0.05 was considered statistically significant.

## 3 Results and Discussion

### 3.1 Establishment of the Radiation‐Induced Premature Aging Mouse Model

We attempted to establish the mouse model of radiation‐induced premature aging. All mice died within about 3 weeks when irradiated once a week (5 Gy per 1w) and within about 7 weeks when irradiated every 2 weeks (5 Gy per 2w), reaching a total radiation dose of 15 Gy in both cases. On the other hand, all mice survived when irradiated every 4 weeks (5 Gy per 4w) and gradually increased body weight, similar to the control (Figure 1B). Two months after initial TBI, the body hair of the mice treated with 5 Gy per 4w turned gray overall (mean gray value: 66.9 ± 5.0 vs. control 28.9 ± 5.5, *p* = 1.99 × 10^−7^), covering about 40% of the body (Figure 1C). Serum levels of inflammation and senescence markers were significantly elevated compared to the control (Figure 1D). Each value is as follows: CDKN2A/p16^INK4a^ (2.87 ± 0.37 vs. control 2.35 ± 0.29 ng/mL, *p* = 0.04068), CRP (20.4 ± 1.66 vs. control 6.94 ± 4.34 ng/mL, *p* = 0.00019), and TNF‐α (310 ± 77.8 vs. control 191 ± 59.2 ng/mL, *p* = 0.02656). Ionizing radiation is a known mutagen and has been used in several studies to induce premature aging in mice. Liao et al. Used a mouse model exposed to 5 Gy of ^60^Co γ‐rays and confirmed aging of hematopoietic stem cells (reduced proliferation/reconstitution ability and biased differentiation to myeloid lineage) 3 months after TBI, which was similar to the characteristics of naturally aged mice (18–24 months of age) [8]. Additionally, melanocyte stem cells present in the bulge region of the hair follicle are preferentially induced to become postmitotic ectopic pigmented melanocytes in response to the aging process, ultimately leading to graying hair [9]. Therefore, this model reproduces the premature aging phenomenon caused by TBI, and the irradiation conditions for mice were decided to be 5 Gy per 4w.

### 3.2 Ameliorative Effect of RP on Radiation‐Induced Premature Aging

We examined the ameliorative effects of RP on aging‐related changes using this radiation‐induced premature aging model. Administration of 10 μg/kg RP once a week or 50 μg/kg RP once every 4 weeks to TBI mice resulted in a similar increase in body weight as controls (Figure 2B). Although no significant changes were observed in the concentrations of serum CDKN2A/p16^INK4a^, CRP, and IFN‐γ after TBI or RP treatment, TNF‐α was significantly decreased by 10 μg/kg RP compared with the TBI group (313 ± 7.27 vs. TBI 360 ± 13.4 pg/mL, *p* < 0.01, Figure 2C). The number of viable BMCs in TBI mice was not increased by RP, suggesting incomplete recovery (Figure 2D). Viable SPCs were similarly decreased after TBI and remained low on day 64, but the recovery significantly occurred in the case of 50 μg/kg RP (5.54 ± 0.88 vs. TBI 3.10 ± 0.81 (×10^7^), *p* < 0.05). It is suggested that hematopoietic stress mobilizes hematopoietic stem/progenitor cells from the bone marrow to the spleen and induces extramedullary hematopoiesis, and hematopoietic function in the spleen is an important and essential factor determining the recovery from radiation‐induced injury [5]. We then focused on SPCs and measured the cellular senescence marker SA‐β‐gal. In abnormal cells where damaged DNA cannot be repaired, cellular senescence occurs, whereby division stops irreversibly, and SA‐β‐gal is overexpressed [10]. TBI increased SA‐β‐gal activity, which was significantly suppressed by 50 μg/kg RP (223 ± 14.6 vs. TBI 260 ± 17.9, *p* < 0.01, Figure 2D). Furthermore, the hair of the TBI mice turned gray overall (mean gray value: 84.1 ± 35.3 vs. Control 42.1 ± 2.19, *p* < 0.05), covering about 66% of the body, which was significantly suppressed by 50 μg/kg RP (*p* < 0.05, Figure 2E). Metformin inhibits radiation‐induced senescence in endothelial cells by promoting DNA repair [11], while Angelica sinensis polysaccharide delays hematopoietic stem cell senescence by regulating sirtuin 1/forkhead box O1 and reducing ROS [12]. In our previous reports [5, 6, 7], RP activates the Nrf2‐Keap1 signaling pathway in SPCs, leading to the induction of target genes involved in redox regulation and antioxidant defense. It enhances cellular redox buffering capacity, suppresses the radiation‐induced ROS accumulation, attenuates oxidative DNA damage through nonhomologous end joining repair, and reduces apoptotic cell death. RP also suppresses the radiation‐induced increase in serum plasminogen activator inhibitor (PAI‐1), a key regulator of inflammation and cellular senescence. These results suggest that RP may also ameliorate radiation‐induced premature aging by sequentially suppressing oxidative stress, DNA damage, and senescence‐associated inflammatory signaling via Nrf2‐mediated antioxidant activation. Notably, even at a dose of 10 μg/kg RP, which is within the clinical range, partial suppression of inflammatory markers was observed, consistent with an effect via these mechanisms. Furthermore, recent studies, supported by epidemiological data from atomic bomb survivors, indicate that ionizing radiation accelerates aging‐related processes such as DNA damage, oxidative stress, inflammation, and stem cell exhaustion, resulting in a systemic aging‐like phenotype [4]. Our mouse model reproduces several of these features, supporting its relevance to radiation‐induced premature aging in humans. Such findings could provide a basis for future research on health issues in aging societies. However, several limitations should be noted. Radiation‐induced senescence may not fully recapitulate all pathways involved in natural aging, and potential systemic toxicity or off‐target effects of repeated RP administration were not evaluated. In addition, the relatively small sample size may limit the robustness of the conclusions. Future studies should include nonirradiated mice treated with RP to clarify the specific effects on aging‐related markers, as well as long‐term follow‐up to assess potential delayed or cumulative adverse effects.

## Author Contributions

M.Y. and I.K. designed and managed the study; M.Y., Y.S., T.H., and I.K. experimented and wrote this manuscript. All authors contributed extensively to discussions regarding the work and the review of the manuscript.

## Funding

This work was supported by a KAKENHI Grant‐in‐Aid for Scientific Research (A) (No. 16H02667 IK).

## Ethics Statement

All procedures related to animal studies were approved by the Animal Research Committee of Hirosaki University (approval number: G17001).

## Conflicts of Interest

The authors declare no conflicts of interest.

## Data Availability

The data that support the findings of this study are available from the corresponding author upon reasonable request.
